# Subcortical contribution to late TMS-induced I-waves in intact humans

**DOI:** 10.3389/fnint.2015.00038

**Published:** 2015-05-27

**Authors:** John Cirillo, Monica A. Perez

**Affiliations:** Department of Physical Medicine and Rehabilitation, Systems Neuroscience Institute, University of PittsburghPittsburgh, PA, USA

**Keywords:** corticospinal volleys, I-wave facilitation, transcranial magnetic stimulation, primary motor cortex, paired-pulse

## Abstract

Paired-pulse transcranial magnetic stimulation (TMS) of the human motor cortex results in consecutive facilitatory motor evoked potential (MEP) peaks in surface electromyography. It has been proposed that early and late MEP peaks involve different mechanisms of action; however, little is known about the characteristics of the later peaks. Using paired-pulse TMS over the hand motor cortex at different test (S1) and conditioning (S2) interstimulus intervals and intensities we examined early (first) and late (second and third) MEP peaks in a resting finger muscle. We demonstrate that the third peak had reduced amplitude and duration compared with the second, regardless of the S1 intensity. Higher S2 intensity increased the amplitude of the third but not the second peak, suggesting that the third peak had a higher threshold. The interval between the second and third peak was longer than between the first and second peak in all conditions even though all peaks had a similar latency dispersion. No differences were found in the amplitude, duration, and threshold of the first and second peaks. A threshold electrical S2 over the cervicomedullary junction facilitated the second and third but not the first peak similarly to TMS. Our results indicate that the third MEP peak is smaller and has higher threshold than the second peak and the similarities between the first and second peak suggest that this is less likely explained by a reduced effectiveness in recruitment. We argue that subcortical pathways might contribute to differences found between late TMS-induced peaks in intact humans.

## Introduction

A single shock over the motor cortex evokes temporally synchronized descending waves in the corticospinal tract in animals and humans (Patton and Amassian, 1954; Di Lazzaro et al., 2012). The earliest wave is due to direct stimulation of the corticospinal neuron at or near the initial segment while later subsequent indirect (I) waves (termed I1, I2, I3, etc.) may arise from transsynaptic activation of corticospinal neurons by intracortical circuits (Di Lazzaro et al., 2012). Transcranial magnetic stimulation (TMS) studies have shown that it is possible to make inferences about the physiology of I-waves from surface electromyography (EMG). Paired-TMS pulses can be precisely timed to increase the amplitude of motor evoked potentials (MEPs) at interstimulus intervals of ~1.5 ms compatible with the I-waves recorded from the epidural space (Tokimura et al., 1996; Ziemann et al., 1998a). It has been proposed that early and late TMS-induced peaks likely involve different mechanisms of action (Di Lazzaro et al., 2012) but little is known about the characteristics of the later MEP peaks.

Previous evidence suggests that there are some differences between later I-waves. For example, the I2-wave has larger amplitude at lower TMS intensity than the I3-wave (Nakamura et al., 1996) and electrical peripheral nerve stimulation suppressed the I2- and I3-wave to a different extent and at a different interstimulus interval (Tokimura et al., 2000). In surface EMG recordings, it is possible to activate circuits responsible for the first and third, but not second, MEP peak in isolation when different TMS coil orientations are used (Day et al., 1989; Sakai et al., 1997). Furthermore, at similar TMS intensities, the second peak is less frequently observed than the third peak (Sakai et al., 1997; Hanajima et al., 1998). Another possible source contributing to differences between the later MEP peaks relates to the origin of these responses. While cortical networks likely contribute to the generation of the first TMS-induced peak (Ziemann et al., 1998b; Ilic et al., 2002), the involvement of subcortical sources for the later TMS-induced peaks cannot be excluded (Tokimura et al., 1996; Ziemann et al., 1998a). Indeed, a recent study demonstrated differences in the characteristics of the second and third MEP peak in individuals with subcortical damage due to spinal cord injury (Cirillo et al., 2015). This may have important implications as later I-waves, specifically the I3, disproportionally contribute to motoneuronal recruitment (Thickbroom, 2011) and are often implicated in long-lasting excitability changes following TMS plasticity interventions (Di Lazzaro et al., 2010). Thus, we hypothesized that the late MEP peaks measured by paired-pulse TMS will differ in their spatial and temporal characteristics, likely involving influences from subcortical networks.

To test our hypothesis, we used paired-pulse TMS over the hand motor cortex at different test (S1) and conditioning (S2) interstimulus intervals and intensities to examine the first, second, and third MEP peak in surface EMG recordings in a resting finger muscle. A mathematical model was used to determine the latency and duration of individual peaks in each subject. Testing was also done using an electrical S2 over the cervicomedullary junction at different intensities. Our findings indicate that spatial and temporal characteristics of the third and second MEP peak differ, likely influenced by subcortical pathways.

## Materials and methods

### Subjects

Fourteen right-handed healthy volunteers (mean age = 43.4 ± 15.8 years, 5 female) participated in the study. All subjects gave written informed consent prior to participation in the study, which was approved by the University of Pittsburgh Research Ethics Committee and in accordance with the guidelines established in the Declaration of Helsinki.

### EMG recordings

EMG was recorded from the right first dorsal interosseous (FDI) muscle through surface electrodes secured to the skin over the muscle belly (Ag–AgCl, 10 mm diameter). The signals were amplified (x 500), filtered (30–1000 Hz), and sampled at 2 kHz (CED 1401 with Signal software, Cambridge Electronic Design, Cambridge, UK) and stored on computer for offline analysis.

### Experimental setup

During testing subjects were seated comfortably in an armchair with their arm flexed 90° at the elbow and the hand resting on a platform with the forearm pronated and the wrist restrained by straps. At the start of the experiment subjects performed 2–3 brief maximal voluntary contractions (MVCs) for 3–5 s with the index finger into abduction separated by 30 s. Verbal feedback by the experimenter and visual feedback of the FDI EMG activity displayed on an oscilloscope was provided throughout the experiment to ensure that subjects remained at rest. A total of 4.6 ± 1.2% trials in which mean rectified EMG activity exceeded ± 2.0 SD of the mean resting EMG, measured 100 ms before the stimulus artifact, were excluded from further analysis (Cirillo et al., 2015).

### TMS

**Figure 1:**
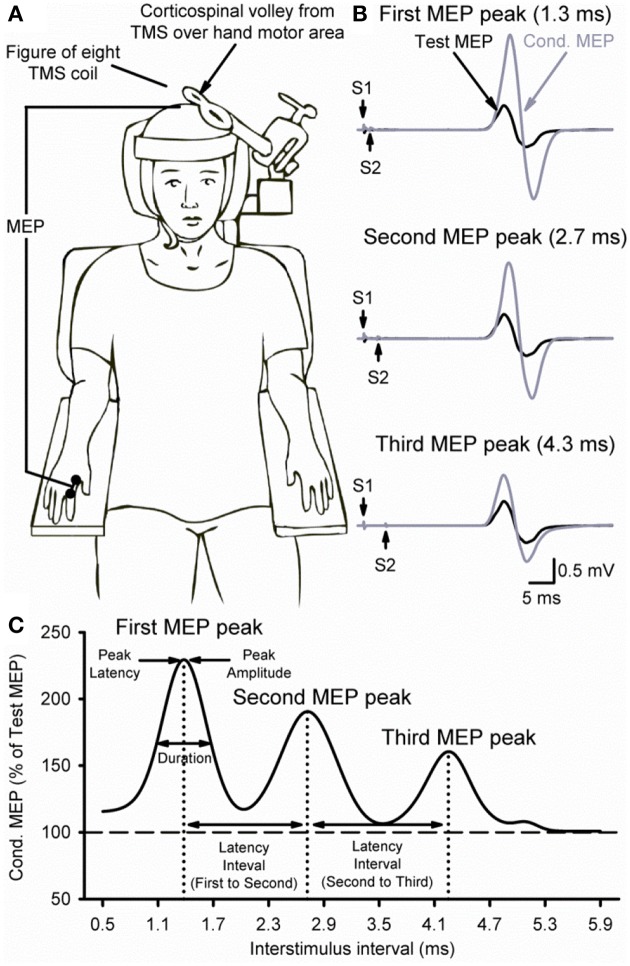
**Experimental setup. (A)** Schematic representation of the experimental setup showing the posture of both hands and TMS coil during testing (illustration). **(B)** Raw MEP traces in the resting first dorsal interosseous muscle at the peak amplitude interval for the first (top trace, 1.3 ms), second (middle trace, 2.7 ms), and third (bottom trace, 4.3 ms) MEP peak. Traces show the average of 20 test (black) and conditioned (gray) MEPs on each condition shown. Arrows indicate the test (S1) and conditioning (S2) stimulus. **(C)** Curve fitting analysis using a three Gaussian model for each MEP peak to estimate individual properties. The vertical dotted lines indicate the latency of each MEP peak localized by the fitting model and the horizontal dashed line represents the size of the Test MEP (baseline). Horizontal arrows indicate the duration and latency interval between MEP peaks.

Transcranial magnetic stimuli were applied using a figure-of-eight coil (loop diameter 70 mm) with two Magstim 200^2^ magnetic stimulators connected with a Magstim Bistim unit (Magstim, Whitland, Dyfed, UK). The coil was held tangentially to the skull with the handle pointing backwards and laterally at an angle of 45° to the sagittal plane. With this coil orientation, current (monophasic waveform) flowed in a posterior-anterior direction (Sakai et al., 1997). The coil was placed at the optimal scalp position for eliciting a motor evoked potential (MEP) in the right FDI muscle. The optimal scalp position was then marked on a cap placed on the head with a pen for reference and the coil firmly secured to the head of the subject by a custom coil holder. To limit head movement, the head of the subject was secured to a headrest by straps (Figure 1A). Single TMS pulses were delivered at 0.2 Hz for all conditions and optimal coil position was continually monitored throughout the experiment. TMS measurements included MEPs, resting motor threshold (RMT), maximal MEP size (MEP-max), and MEP peaks (first, second, and third).

### MEPs

RMT (52.6 ± 12.9%) was determined as the minimum stimulus intensity required to elicit an MEP in the relaxed FDI of at least 50 μV in amplitude in 5 out of 10 consecutive trials and expressed relative to the maximum stimulator output (MSO; Rothwell et al., 1999). The MEP-max (4.61 ± 2.8 mV) was defined by increasing the stimulus intensity in 5% increments of MSO in the relaxed FDI until the MEP amplitude did not show any additional increase.

### MEP peaks

TMS-induced MEP peaks were assessed at rest using a previously described paired-pulse TMS paradigm (Tokimura et al., 1996; Ziemann et al., 1998a). A test stimulus (S1) was set to produce a MEP amplitude of ~1 mV (0.97 ± 0.31 mV; 120% of RMT) when given alone at rest, whereas the conditioning stimulus (S2) was set to 90% of RMT. The S1 elicited a test MEP and the S2 elicited a conditioned MEP (Figure 1B). For all conditions, the S2 was delivered at interstimulus intervals (ISIs) of 0.5–5.9 ms (tested in 0.2 ms steps, 28 intervals) after the S1. MEPs at each ISI were tested twice with the order of presentation randomized and each time 10 MEPs were collected. Because the size of the conditioned MEP is influenced by the intensity of the S1 (Ziemann et al., 1998a; Wagle-Shukla et al., 2009; Shirota et al., 2010) in a control experiment MEP peaks were tested by using a higher (~3 mV, 140% of RMT) and a lower (~0.05 mV, 100% of RMT) S1 while keeping the S2 set to 90% of RMT. The intensity of the S2 also influences the size of the conditioned MEP (Hanajima et al., 2002; Shirota et al., 2010). Therefore, an additional control experiment was performed where we examined the third MEP peak (between 3.7 and 5.1 ms), as well as the first (1.3 ms) and second (2.7 ms) peak with a suprathreshold (105% of RMT) and a subthreshold (80% of RMT) S2 while using 120% of RMT for the S1. MEP peaks were calculated by expressing the size of the conditioned MEP as a percentage of the size of the test MEP [(conditioned MEP × 100)/(test MEP)].

MEP peaks were also assessed using a S1 elicited by TMS and a S2 elicited by electrical stimulation over the cervicomedullary junction at ISIs of 1.3 ms (first), 2.7 ms (second), and 4.3 ms (third) in the resting FDI muscle. Cervicomedullary junction stimulation was applied by a high-voltage electrical current (100-μs duration; DS7AH Digitimer) passed between adhesive Ag-AgCl electrodes fixed to the skin behind the mastoid process (Ugawa et al., 1992; Taylor and Gandevia, 2004). The intensity of the S1 was set at 120% of RMT, while the electrical S2 was set to 80% (≤ 20 μV), 100% (20–100 μV), or 105% (100–500 μV) of cervicomedullary motor evoked potential (CMEP) threshold. Each ISI was adjusted to account for the difference in MEP onset latency between M1 and cervicomedullary level (4.70 ± 0.97 ms). Ten MEP trials were collected at each ISI, with 40 trials collected in each condition.

### MEP peaks analysis using a three Gaussian model

We fitted our data into a three Gaussian model (Thickbroom, 2011; Delvendahl et al., 2014; Cirillo et al., 2015) to accurately estimate the peak latency and duration of each peak in each subject (Figure 1C). For each peak *i* with a given latency *t*_*i*_, amplitude *A*_*i*_ and width (Gaussian sigma) σ_*i*_, and overall baseline *y*_0_, and small-ISI baseline *y*_0,*L*_, peaks were modeled as

$$\begin{array}{l} I_{1}(t)=\{\begin{array}{ll} y_{0,L}+(A_{1}-y_{0,L})*e^{-\frac{{\left(t-t_{1}\right)}^{2}}{2\sigma_{1}{}^{2}}} & t<t_{1}\\ A_{1}*e^{-\frac{{\left(t-t_{1}\right)}^{2}}{2\sigma_{1}{}^{2}}} & t\geq t_{1} \end{array}\\ I_{2}(t)=A_{2}*e^{-\frac{{\left(t-t_{2}\right)}^{2}}{2\sigma_{2}{}^{2}}}\\ I_{3}(t)=A_{3}*e^{-\frac{{\left(t-t_{3}\right)}^{2}}{2\sigma_{3}{}^{2}}}\\ y(t)=100+I_{1}(t)+I_{2}(t)+I_{3}(t)+y_{0} \end{array}$$

where *y* is the peak amplitude (see MEP peaks section for calculation) and *t* is the ISI. Data was fitted to the model for each subject using a 1000-iteration bootstrapping procedure (Efron and Tibshirani, 1993; DiCiccio and Efron, 1996) using the MATLAB bootci function. On each iteration, a data set was created by sampling individual normalized MEPs with replacement. A curve fit was then performed using a trust region reflective least squares fit algorithm (Coleman and Li, 1996). Parameter estimates for each subject were chosen as the mean of the 1000 fits, and 95% confidence intervals (CI) were computed across this sample. All peaks where amplitudes had CIs not inclusive of 0 were deemed significant and included in the group analyses.

### Data analysis

Normal distribution was tested by the Shapiro-Wilk's test and homogeneity of variances by the Levene's test of equality and Mauchly's test of sphericity. When normal distribution could not be assumed data was log transformed. When sphericity could not be assumed the Greenhouse-Geisser correction statistic was used. A One-Way repeated measures ANOVA was performed to determine the effect of ISI (0.5–5.9 ms, in 0.2 ms steps) on the amplitude of the conditioned MEP. The same analysis was also performed to determine the differences across PEAKS (first, second, and third) on latency and duration. A Two-Way repeated measures ANOVA was used to determine the effect of TRIAL (Trial 1, Trial 2) and ISI on the amplitude of the conditioned MEP and PEAKS latency and duration. A Two-Way repeated measures ANOVA was also used to determine the effects of S1 INTENSITY (100, 120, and 140% of RMT), S2 INTENSITY (80, 90, and 105% of RMT), electrical S2 INTENSITY (80, 100, and 105% of RMT) and ISIs on the amplitude of the conditioned MEP and PEAKS latency and duration. A *post-hoc* Bonferroni test was used to test for significant comparisons. Pearson correlation analysis was used as needed. Significance was set at *P* < 0.05. Group data are presented as means ± SD in the text.

## Results

### MEP peaks tested at different S1 intensities

**Figure 2:**
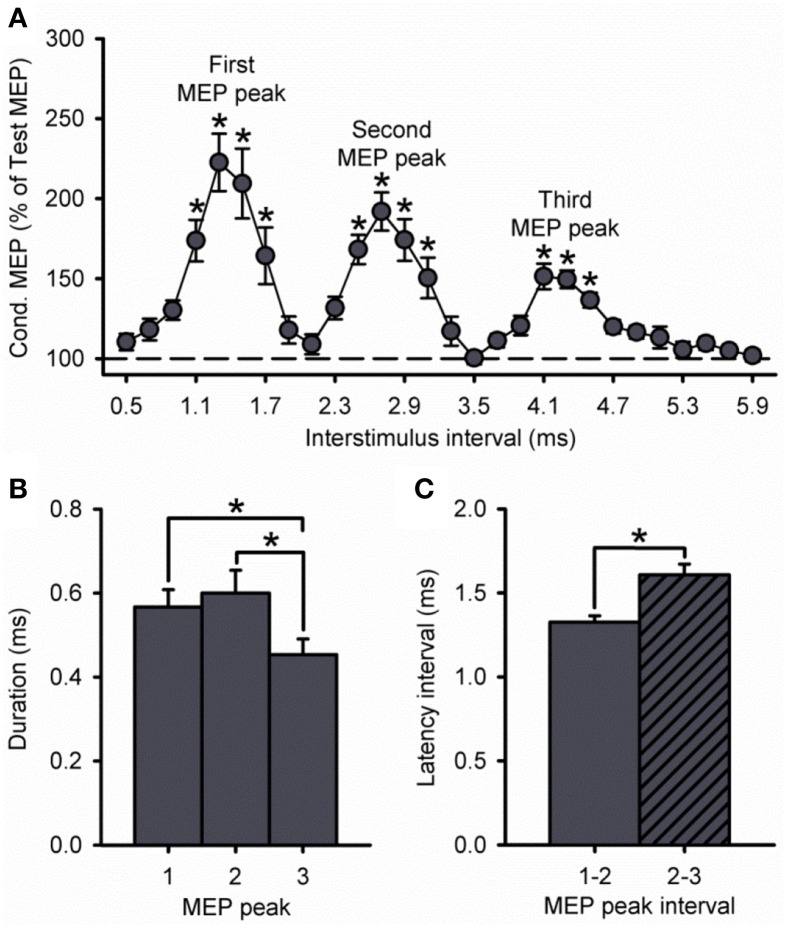
**MEP peaks. (A)** Group data showing peaks tested by paired-pulse TMS with a S1 intensity of 120% of RMT and S2 set to 90% of RMT (*n* = 14). The abscissa shows the ISIs tested (0.5–5.9 ms, in 0.2 ms steps). The ordinate shows the size of the conditioned MEP (expressed as a % of the Test MEP, horizontal dashed line). Note that the conditioned MEP was largely facilitated at stimulus intervals corresponding to the I1, I2, and I3 waves from epidural recordings. **(B)** Group data showing the duration of each MEP peak extracted from individual curve fit parameters. The abscissa shows each MEP peak (first = 1, second = 2, and third = 3). The ordinate shows the duration (in milliseconds). Note that duration was decreased for the third MEP peak compared with the first and second peak. **(C)** Group data showing the latency interval between MEP peaks extracted from individual curve fit parameters. Note that the interval between the second and third peaks (diagonally hatched bars) was prolonged compared with the interval between the first and second peaks (solid filled bars). Error bars indicate SEs. ^*^P < 0.05.

Figure 1B illustrates examples of test (black traces) and conditioned (gray traces) MEPs in the resting FDI muscle from a representative subject using an S1 of 120% of RMT and an S2 of 90% of RMT. Note that the amplitude of the conditioned MEP at 4.3 ms increased to a lesser extent than at 2.7 and 1.3 ms interstimulus intervals. At this intensity, repeated measures ANOVA revealed a significant effect of ISI [*F*_(3, 43)_ = 15.6, *P* < 0.001; Figure 2A] on the conditioned MEP amplitude. *Post-hoc* testing indicate that the amplitude of the conditioned MEP was increased at intervals corresponding to the first peak from 1.1 to 1.7 ms (*P* < 0.01), second peak from 2.5 to 3.1 ms (*P* < 0.01), and third peak from 4.1 to 4.5 ms (*P* = 0.04). A group analysis showed that amplitudes were similar across repeated trials [*F*_(1, 13)_ = 0.2, *P* = 0.64]. Our Gaussian model analysis showed that the duration of the third peak (0.45 ± 0.14 ms) was decreased compared with the second (0.60 ± 0.20 ms, *P* = 0.03) and first (0.57 ± 0.15 ms, *P* = 0.03; Figure 2B) peak. Here, we observed that 11/14 subjects showed a decrease in the third peak duration compared with the other peaks. No differences were found between the duration of the first and second peak (*P* = 0.29). The latency of the first (1.37 ± 0.10 ms), second (2.70 ± 0.14 ms) and third (4.31 ± 0.26 ms) peak were significantly different [*F*_(2, 39)_ = 964.1, *P* < 0.001]. The interval between the latency for the second and third peak (1.61 ± 0.24 ms) was prolonged compared with the interval between the first and second peak (1.33 ± 0.14 ms; *P* < 0.01; Figure 2C).

**Figure 3:**
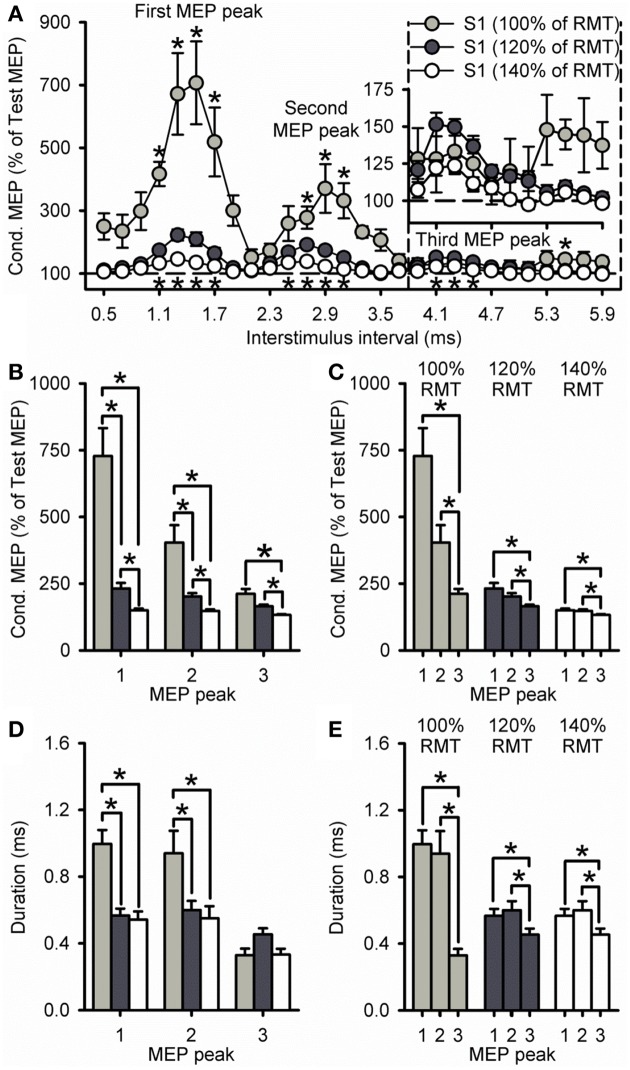
**Effect of different S1 intensities on MEP peaks. (A)** Group data showing peaks tested by paired-pulse TMS at S1 intensities of 100% (light gray circles, *n* = 7), 120% (dark gray circles, *n* = 14), and 140% (open circles, *n* = 10) of RMT. The abscissa shows the ISIs tested (0.5–5.9 ms, in 0.2 ms steps). The ordinate shows the size of the conditioned MEP (expressed as a % of the Test MEP, horizontal dashed line). The S2 was set to 90% of RMT for all conditions. Graphs showing the group data for the size of the conditioned MEP as a % of the test MEP **(B,C)** and the duration **(D,E)** of each MEP peak extracted from individual curve fit parameters. The abscissa shows each MEP peak (first = 1, second = 2, and third = 3). The ordinate shows the size of the conditioned MEP (expressed as a % of the Test MEP) and duration (in milliseconds). Note that the amplitude of all peaks was reduced at an S1 of 140% of RMT and increased at an S1 of 100% RMT. Also note that amplitude and duration were decreased for the third MEP peak compared with the first and second peak for all S1 intensities. Error bars indicate SEs. ^*^P < 0.05.

Because the size of the conditioned MEP is influenced by the intensity of the S1, Figure 3A shows data using an S1 of 100, 120, and 140% of RMT. A group analysis showed a significant effect of ISI [*F*_(7, 195)_ = 35.8, *P* < 0.001], S1 INTENSITY [*F*_(2, 28)_ = 43.1, *P* < 0.001] and in their interaction [*F*_(14, 195)_ = 9.5, *P* < 0.001] on the amplitude of the conditioned MEP. *Post-hoc* testing revealed that the conditioned MEP was reduced at 140% compared with 120% at ISIs corresponding to the first (1.1 to 1.7 ms; *P* = 0.02), second (2.5 to 3.1 ms; *P* = 0.01), and third (4.1 to 4.5 ms; *P* < 0.01; Figure 3B) MEP peak. In contrast, the conditioned MEP was increased at 100% compared with 120% at ISIs corresponding to the first (1.1 to 1.7 ms, *P* < 0.01), second (2.7 to 3.1 ms, *P* = 0.04), and third (5.5 ms, *P* < 0.01; Figure 3B) MEP peak. A comparison within S1 intensities showed that the amplitude of the third peak was reduced compared with the first and second peak for an S1 of 100% (first = 753 ± 325%, second = 404 ± 172%, third = 194 ± 42%; *P* = 0.04), 120% (first = 232 ± 78%, second = 202 ± 47%, third = 165 ± 23%; *P* = 0.01), and 140% (first = 151 ± 19%, second = 148 ± 18%, third = 133 ± 10%; *P* = 0.03; Figure 3C) of RMT. The amplitude for the first and second peak remained similar for each S1 intensity (100%, *P* = 0.12; 120%, *P* = 0.24; 140%, *P* = 0.98).

Our curve fitting analysis showed that when comparing the duration of the peaks between S1 intensities, the first and second, but not third MEP peak were increased for 100% compared with 120% (first peak, *P* < 0.001; second peak, *P* = 0.03; third peak, *P* = 0.14) and 140% (first peak, *P* < 0.001; second peak, *P* = 0.04; third peak, *P* = 0.99; Figure 3D). The duration of all peaks was similar between 120 and 140% (*P* = 0.29; Figure 3D). We also found that the duration of the third MEP peak was decreased compared with the first and second MEP peak when using a S1 of 100% (third peak = 0.33 ± 0.11 ms, second peak = 0.94 ± 0.36 ms, first peak = 1.00 ± 0.22 ms;7/7 subjects, *P* = 0.02), 120% (third peak = 0.45 ± 0.14 ms, second peak = 0.60 ± 0.20 ms, first peak = 0.57 ± 0.15 ms; 11/14 subjects, *P* = 0.03), and 140% (third peak = 0.33 ± 0.11 ms, second peak = 0.55 ± 0.23 ms, first peak = 0.54 ± 0.15 ms; 9/10 subjects, *P* = 0.01; Figure 3E). The duration of the first and second peak remained similar (100%, *P* = 0.96; 120%, *P* = 0.90; 140%, *P* = 0.99; Figure 3E). Curve fitting analysis also showed that the latency of the first, second and third MEP peak was similar for S1 intensities of 120 and 140%, but changed when the S1 was set at 100% of RMT (Table 1). Importantly, a group analysis showed that the duration [100%, *F*_(1, 6)_ = 2.7, *P* = 0.17; 120%, *F*_(1, 13)_ = 0.2, *P* = 0.64, *P* = 0.91; 140%, *F*_(1, 9)_ = 0.6, *P* = 0.45] and latency [100%, *F*_(1, 6)_ < 0.1, *P* = 0.92; 120%, *F*_(1, 13)_ < 0.1, *P* = 0.91; 140%, *F*_(1, 9)_ = 0.7, *P* = 0.42] of all peaks were similar across repeated trials for all S1 intensities.

**Table 1:** **Latency of MEP peaks**.

**MEP Peak**	**100% of RMT**	**120% of RMT**	**140% RMT**	***P*-value**
1	1.38 ± 0.10	1.37 ± 0.10	1.33 ± 0.11	0.56
2	3.06 ± 0.17	2.70 ± 0.14	2.68 ± 0.20	0.03
3	5.02 ± 0.40	4.31 ± 0.26	4.25 ± 0.24	<0.001

*Values are mean ± SD of MEP peak latency for test stimulus (S1) intensities of 100%, 120%, and 140% of resting motor threshold (RMT) in the first dorsal interosseous (FDI) muscle. P-values represent ANOVA tests performed across S1 intensities on each MEP peak. Note that latency was prolonged for MEP peaks 2 and 3 at 100% compared with 120% and 140% of RMT. Also note that the latency of each MEP peak was different at all S1 intensities*.

### MEP peaks tested at different S2 intensities

**Figure 4:**
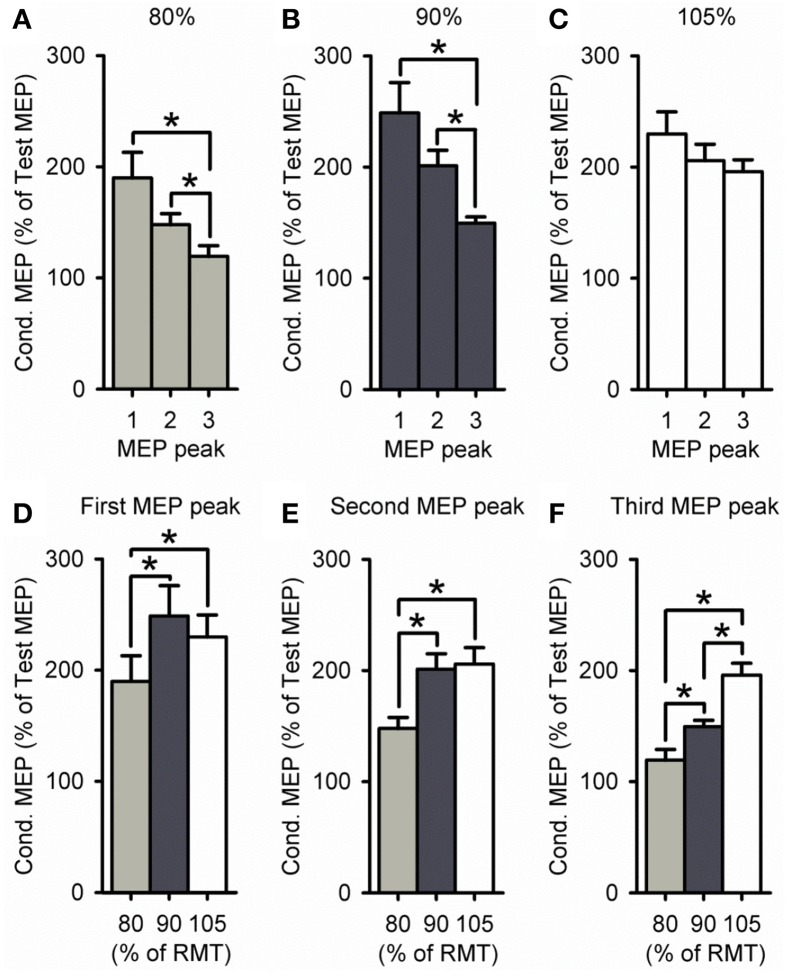
**Effect of different S2 intensities on MEP peaks. (A–F)** Group data showing the first (1.3 ms), second (2.7 ms), and third (4.3 ms) MEP peak tested by paired-pulse TMS at S2 intensities of 80% (light gray bars, *n* = 9), 90% (dark gray bars, *n* = 14), and 105% (open bars, *n* = 9) of RMT. The abscissa shows the MEP peak (first = 1, second = 2, and third = 3; **A–C**) or S2 intensity (80, 90, and 105% of RMT; **D–F**). The ordinate shows the size of the conditioned MEP (expressed as a % of the Test MEP). Note that amplitude was similar for all MEP peaks for an S2 of 105% of RMT, whereas the third peak was reduced compared with the first and second for S2 intensities of 80–90% of RMT. Also note that the amplitude of the third peak was increased for an S2 of 105% of RMT, while the amplitude was decreased for all MEP peaks for an S2 of 80% of RMT. Error bars indicate SEs. ^*^P < 0.05.

A group analysis showed a significant effect of ISI [*F*_(2, 60)_ = 21.2, *P* < 0.001], S2 INTENSITY [*F*_(2, 30)_ = 8.8, *P* < 0.01] and in their interaction [*F*_(4, 60)_ = 2.7, *P* = 0.04; Figure 4] on the amplitude of the conditioned MEP. *Post-hoc* testing showed that for a S2 intensity of 105% the amplitude of the third peak (198 ± 27%) was similar to the first (218 ± 58%, *P* = 0.57) and second (210 ± 43%, *P* = 0.94; Figure 4C). In contrast, the amplitude of the third peak was reduced compared with the first and second peak for an S2 of 90% (*P* < 0.01; Figure 4B) and 80% (*P* = 0.02; Figure 4A). A comparison across S2 intensities showed that the amplitude of the third peak was increased for 105% compared with 90% (*P* < 0.01) and 80% (*P* < 0.001; Figure 4F). In contrast, the amplitude was similar between 105 and 90% of RMT for the first (*P* = 0.13; Figure 4D) and second (*P* = 0.36; Figure 4E) peak. All together these results indicate that the third peak had a higher threshold than the second and first peak. Also note that the amplitude of MEP peaks was reduced for the S2 intensity of 80% compared with 105 and 90% of the RMT for all peaks (first, *P* = 0.01; second, *P* = 0.02; and third, *P* = 0.01).

### MEP peaks latency distribution

**Figure 5:**
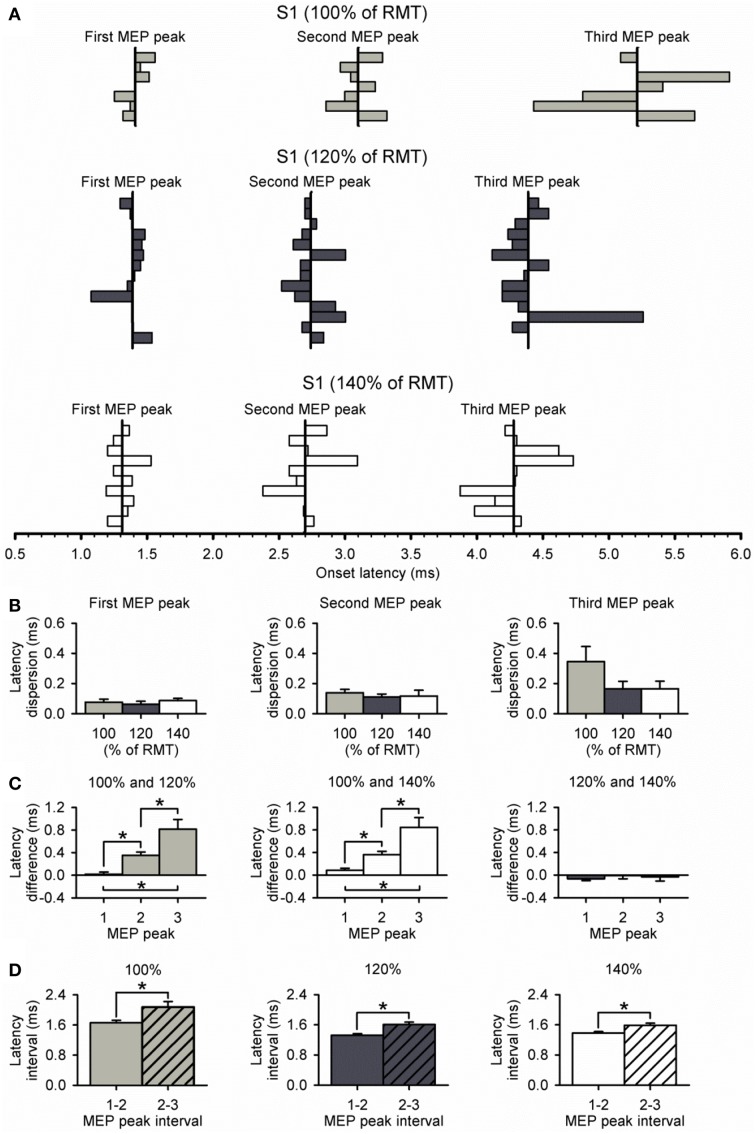
**Dispersion of MEP peaks latency. (A)** Latency of each MEP peak and its dispersion at S1 intensities of 100% (light gray bars, *n* = 7), 120% (dark gray bars, *n* = 14), and 140% (open bars, *n* = 10) of RMT. The vertical line indicates the mean latency for each peak in all subjects at each S1 intensity. **(B)** Graphs showing the group data for relative dispersion of MEP peak latencies at different S1 intensities. Note that dispersion was similar between each MEP peak and S1 intensity. **(C)** Graphs showing the group data for latency differences across S1 intensities for each MEP peak. Note that latencies of MEP peaks progressively prolonged when using a low S1 intensity of 100% of RMT. Also note that the latencies of each MEP peak were similar when using S1 intensities of 120% and 140% of RMT. **(D)** Graphs showing the group data for latency interval between MEP peaks for each S1 intensity. Note that the interval between the second and third peak (diagonally hatched bars) was prolonged compared with the interval between the first and second peak (solid filled bars) for all S1 intensities. Error bars indicate SEs. ^*^P < 0.05.

Figure 5A shows the latency of each MEP peak for individual subjects at all S1 intensities tested. We found that the dispersion of latencies was similar for each peak across S1 intensities (first peak, *P* = 0.74; second peak, *P* = 0.77; third peak, *P* = 0.24; Figure 5B). Furthermore, the dispersion of the third peak was similar to the second and first, regardless of the S1 intensity tested (100%, *P* = 0.11; 120%, *P* = 0.27; 140%, *P* = 0.26). Notably, the difference in latency for the third peak was more prolonged than the second and first when a S1 intensity of 100% was compared with 120% (*P* < 0.01) and 140% (*P* = 0.01; Figure 5C) of RMT. No differences were observed in the latency for all peaks when an S1 of 120 and 140% of RMT was used (*P* = 0.48; Figure 5C). When calculating the interval between MEP peaks from latency estimations, we found that the interval between the second and third peak was prolonged compared with the first and second peak when a S1 of 100% (first-second = 1.66 ± 0.17 ms, second-third = 2.07 ± 0.39 ms; *P* = 0.04), 120% (first-second = 1.33 ± 0.14 ms, second-third = 1.61 ± 0.24 ms; *P* < 0.01), and 140% (first-second = 1.38 ± 0.12 ms, second-third = 1.59 ± 0.17 ms; *P* < 0.01; Figure 5D) of RMT was used.

### Cervicomedullary junction stimulation as the S2

**Figure 6:**
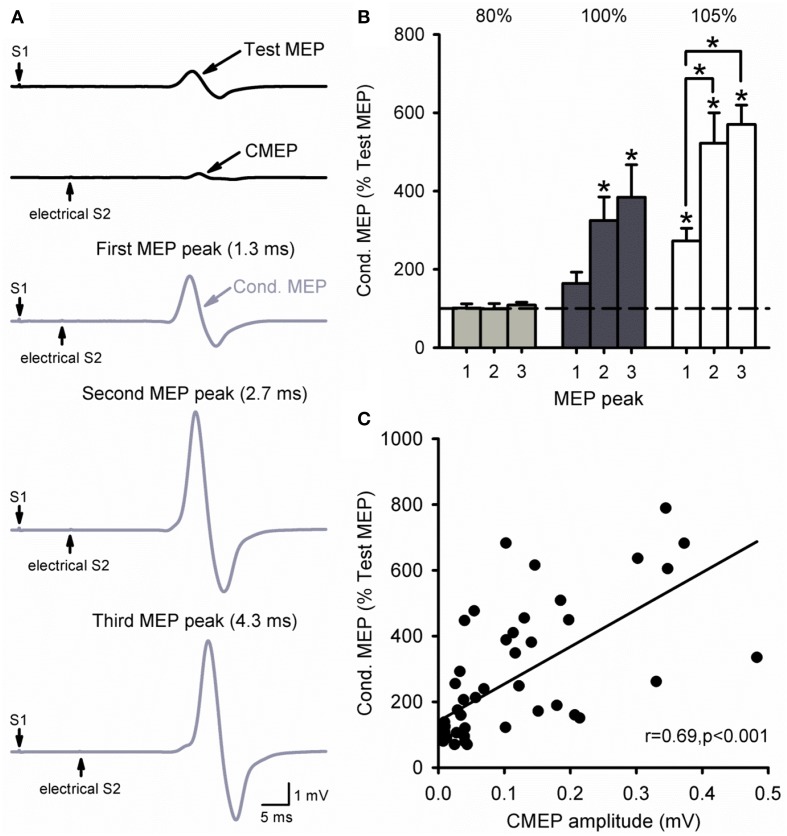
**Cervicomedullary junction stimulation as the S2. (A)** Raw MEP traces in the resting first dorsal interosseous muscle for the test (S1) and conditioning (S2) stimulus (black) at the first (1.3 ms), second (2.7 ms), and third (4.3 ms) MEP peak intervals (gray). Traces show the average of 10 responses on each condition shown. Arrows indicate the test (S1) and conditioning (electrical S2) stimulus. **(B)** Graphs showing the group data (*n* = 6) for MEP amplitude at each MEP peak ISI. The abscissa shows the intensity of the electrical S2 (below threshold, threshold, above threshold). The ordinate shows the size of the conditioned MEP (expressed as a % of the Test MEP). Note the increased MEP amplitude for the second and third peaks compared with the first at an electrical S2 set to threshold or above. **(C)** Correlation analysis between the size of the CMEP and conditioned MEP. The abscissa shows the CMEP amplitude (mV) and the ordinate shows the size of the conditioned MEP (expressed as a % of the Test MEP). Note that there was a positive correlation between the size of the CMEP and that of the conditioned MEP. Error bars indicate SEs. ^*^P < 0.05.

Figure 6A illustrates MEP recordings from a representative subject where threshold electrical S2 was delivered at the cervicomedullary junction following a TMS S1 of 120% of RMT. Note that the amplitude of the conditioned MEP was larger at ISIs of 2.7 ms (second peak) and 4.3 ms (third peak) compared with 1.3 ms (first peak). The group data (Figure 6B) shows that there was no facilitation of the MEP compared with baseline when the electrical S2 was below threshold [80%, *F*_(3, 12)_ = 0.3, *P* = 0.81]. In contrast, the amplitude of the conditioned MEP was facilitated compared with baseline when the electrical S2 was at threshold (100%) for ISIs of 2.7 ms (325 ± 144%, *P* = 0.01) and 4.3 ms (384 ± 186%, *P* < 0.01), but not 1.3 ms (164 ± 65%, *P* = 0.34). When the electrical S2 was above threshold (105%), the conditioned MEP was facilitated at all ISIs compared with baseline (1.3 ms: 273 ± 72%, *P* < 0.01; 2.7 ms: 522 ± 174%, *P* < 0.001; 4.3 ms: 570 ± 110%, *P* < 0.001). We also found that the amplitude of the CMEP positively correlated to the amplitude of the conditioned MEP (Figure 6C). Note that individuals had an increased conditioned MEP when the amplitude of the CMEP was increased.

## Discussion

The present study examined late TMS-induced MEP peaks in intact humans. Using paired-pulse TMS over the hand motor cortex at different test (S1) and conditioning (S2) interstimulus intervals and intensities we demonstrate that the third MEP peak had a reduced amplitude and duration compared with the second peak, regardless of the S1 intensity. Higher S2 intensity increased the amplitude of the third peak but not the second peak, suggesting that the third peak had a higher threshold. The interval between the second and third peak was longer than between the first and second peak in all conditions even though all peaks had a similar latency dispersion. No differences were found in the amplitude, duration, and threshold of the first and second peak. A threshold electrical S2 over the cervicomedullary junction facilitated the second and third but not the first peak similarly to TMS. We demonstrate that the third MEP peak is smaller and has a higher threshold than the second peak which are likely influenced by contributions from subcortical pathways.

### Characteristics of late TMS-induced MEP peaks

Our findings indicate that differences exist in the characteristics of the second and third TMS-induced MEP peaks. First, we found that the amplitude of the third peak was reduced compared with the second peak. Although no previous studies have systematically looked at differences in the amplitude across I-waves some results point in the same direction. For example, the size of the I3-wave recorded from the epidural space (Di Lazzaro et al., 1998a) seems to be reduced compared with the earlier I-waves. Similarly, the size of the third MEP peak recorded by surface EMG appears to be of a lesser amplitude compared with earlier responses (Ziemann et al., 1998a). The recruitment and amplitude of I-waves recorded from the epidural space and peristimulus time histograms of single motor units is influenced by the TMS intensity (Day et al., 1989; Nakamura et al., 1996; Sakai et al., 1997; Di Lazzaro et al., 1998b); therefore, a possibility is that the third peak has a higher threshold compared with the second peak. This is consistent with a previous study showing that the I2-wave recorded from the epidural space could be elicited by using a lower stimulus intensity compared with the I3 (Nakamura et al., 1996). Also, previous evidence from epidural recordings shows that the I3-wave can be elicited at higher stimulus intensities compared with the earlier I-waves (Di Lazzaro et al., 1998b). This possibility is also supported by our results showing that the amplitude of the third but not the second MEP peak was increased when a higher conditioning stimulus intensity was used. We also found that the duration of the third peak was reduced compared with the second peak. Notably, with higher and lower test stimulus intensities the third peak continued to have reduced amplitude and duration compared with the second peak, suggesting that these differences were not an epiphenomenon of the stimulus intensity. An important question is if the smaller size and higher threshold of the third peak compared with the preceding peaks is the result of a reduced effectiveness in recruitment. Our results indicate that it is less likely that this was the case. On one side, we found that the amplitude and duration of the first and second peak were similar regardless of the stimulus intensity tested. If a decrease in the efficacy of recruitment contributed to our results we would have expected that the characteristics of the second compared with the first peak would also be affected, which was not the case. On the other side, we found that the interval between the second and third peak was longer than between the first and second peak in all conditions tested. This agrees with the results from epidural recordings showing that the interval between I-waves is not identical (Kernell and Chien-Ping, 1967). Also, MEP peaks recorded from surface EMG electrodes have been reported at a longer range of intervals for the third compared with the second MEP peak (Ziemann et al., 1998a). It is possible that the longer delay in latency between later peaks results from an increased variability in their recruitment threshold (Kernell and Chien-Ping, 1967). However, this is less likely in our data since we found that the dispersion of latencies was similar for all peaks at all stimulus intensities tested. Indeed, we found that the third peak was delayed to a larger extent than the second peak at lower test TMS stimulus intensities, suggesting that other factors contributed to our results. Thus, our results indicate that the third MEP peak is smaller and has a higher threshold than the second peak with similarities between the first and second peak suggesting that it is less likely that these differences can be explained by a reduced effectiveness in recruitment.

### Mechanisms of TMS-induced late MEP peaks

Evidence from pharmacological studies in humans suggested that GABAergic neuronal circuits are involved in the generation of TMS-induced later MEP peaks (Ziemann et al., 1998b; Ilic et al., 2002). A paired-pulse TMS paradigm examining intracortical inhibition suppresses the size of the later peaks (Nakamura et al., 1997; Di Lazzaro et al., 1998b). Also, when intracortical inhibition is measured in combination with the MEP peaks, using a triple pulse stimulation technique, the later peaks are facilitated (Wagle-Shukla et al., 2009), supporting the contribution from intracortical circuits.

Our results demonstrate that the second and third MEP peak were facilitated following a threshold electrical stimulus applied at the cervicomedullary junction, which is unlikely to activate intracortical circuits, suggesting that subcortical mechanisms are also likely to contribute to the generation of these later peaks. This agrees with recent results showing distinct and pronounced deficits in the later TMS-induced peaks in individuals with subcortical damage due to incomplete spinal cord injury (Cirillo et al., 2015). Indeed, it might not be surprising that later MEP peaks measured by surface EMG will be affected by subcortical influences. First, evidence showed that cortical and spinal influences can both contribute to the changes in MEP size (Burke and Pierrot-Deseilligny, 2010), which are used to noninvasively assess these peaks. Second, the later peaks disappeared during voluntary activity, which can be explained by a subcortical involvement (Ziemann et al., 1998a). The only study that tested the effect of an electrical pulse on the later peaks showed mixed results (Tokimura et al., 1996). Others have examined the first MEP peak during small levels of voluntary contraction or at rest, showing no facilitation (Tokimura et al., 1996; Ziemann et al., 1998a) or an increased (Chen and Garg, 2000) first MEP peak amplitude, respectively, when different stimulus intensities were used. In agreement, we found that a larger size of the electrically evoked CMEP was associated with a larger facilitation in all peaks. When we increased the S2 electrical stimulus intensity above threshold (105%) all peaks were facilitated. The facilitation present in the first peak at this higher stimulus intensity might be related to the recruitment of non-refractory axons (Tokimura et al., 1996), which will affect the summation of EPSPs at the spinal motoneurone pool. The larger facilitation of the second and third peak compared with the first peak, at this suprathreshold intensity, also supports the view that the facilitation of the later peaks is related to excitation of different neuronal elements than the first peak.

The next intriguing question is what is/are the possible neuronal pathway(s) that contributed to the later MEP peaks? A possibility is that different subcortical circuits were involved. For example, evidence showed that spinally-mediated disynaptic reciprocal Ia inhibition (Crone et al., 2004) and recurrent inhibition (Mazzocchio et al., 1994) can be affected by corticospinal influences. Reciprocal inhibition is present at ISIs of 2–3 ms and recurrent inhibition is present at ISIs of 5–8 ms (Katz and Pierrot-Deseilligny, 1999; Crone et al., 2004), which closely correspond to the intervals at which the second and third peak are observed and might have affected our results. Another possibility is that activity in intracortical pathways also contributed to our results. Modeling studies have proposed that the summation of EPSPs and IPSPs on distal synapses on corticospinal neurons influence later I-waves (Rusu et al., 2014). Indeed, evidence showed that intracortical inhibition is more prominent at intervals targeting the third compared with the second MEP peak (Nakamura et al., 1997; Di Lazzaro et al., 1998b). It is also possible that inputs arriving from cortico-cortico afferents contributed to our results because later peaks may reflect activity from other cortical areas (Amassian et al., 1987; Rothwell, 1991; Edgley et al., 1997; Groppa et al., 2011). Regardless of the specific mechanisms contributing to our effects in the later peaks, for the first time our findings demonstrate that subcortical pathways contribute to modulate later TMS-induced MEP peaks in human subjects.

### Functional considerations

Although the biological relevance of the TMS-induced peaks remains unclear a possibility is that these peaks represent a route for examining the summation of multiple synaptic inputs (Ziemann and Rothwell, 2000). It has been reported that changes in the late MEP peaks can reflect some information about aspects of an upcoming movement (Cattaneo et al., 2005; Prabhu et al., 2007). Thus, our results showing an involvement of subcortical influences in the generation of these later TMS-induced peaks may open new targets for protocols aiming to change synaptic plasticity (Thickbroom et al., 2006; Cash et al., 2009). This might be particularly relevant for individuals with incomplete spinal cord injury in whom the temporal and spatial characteristics of the late peaks correlates with MEP size and aspects of hand voluntary motor output (Cirillo et al., 2015).

### Conflict of interest statement

The authors declare that the research was conducted in the absence of any commercial or financial relationships that could be construed as a potential conflict of interest.
